# Association of Vegetable Consumption with Stroke in China: A Longitudinal Study

**DOI:** 10.3390/nu15071583

**Published:** 2023-03-24

**Authors:** Meiru Lv, Chang Su, Feifei Huang, Huijun Wang, Zhihong Wang, Bing Zhang, Wenwen Du

**Affiliations:** 1 National Institute for Nutrition and Health, Chinese Center for Disease Control and Prevention, Beijing 100050, China; 2 Key Laboratory of Trace Element Nutrition of Health Commission of China, Beijing 100050, China

**Keywords:** vegetable consumption, dark vegetables, stroke, Chinese population

## Abstract

Research on the relationship between vegetable consumption and stroke among the Chinese population is still rare. This study aimed to explore the association between vegetable consumption and stroke. Using data from the China Health and Nutrition Survey (1991–2018), we included 15,145 participants over 40 years old without stroke as the baseline. Participants were categorized into five groups according to vegetable consumption. The adjusted HRs of stroke associated with vegetable consumption were calculated using the COX proportional hazards model. During the follow-up, 504 stroke cases were detected (303 men and 201 women). For the females, compared with the Q1 group of vegetable consumption, the multivariable-adjusted HRs for stroke were 0.60 (95%CI 0.36, 1.00) in the group of Q4. No significant associations between vegetable consumption and stroke were found among males. Furthermore, compared with the Q1 group of dark vegetable consumption, for the whole subjects, the multivariable-adjusted HRs for stroke were 0.68 (95%CI 0.50, 0.92) in the group of Q4. For the females, compared with the Q1 group of dark vegetable consumption, the multivariable-adjusted HRs for stroke were 0.49 (95%CI 0.30, 0.80) in the group of Q4. In conclusion, this study suggested that vegetable consumption decreases the risk of stroke among Chinese females. In addition, the intake of dark vegetables was inversely associated with stroke.

## 1. Introduction

Stroke has become one of the diseases that seriously affect human life and health around the world. Stroke is the second-leading cause of death and the third-leading cause of death and disability combined in the world [1]. In France, 140,000 people were hospitalized each year for stroke, and ischemic stroke is the most common type [2]. Stroke was still the fifth leading cause of death in the United States, although the incidence of stroke has declined over the past decade [3]. In Italy, with 130,000 new strokes per year in the general population, ischemic stroke was the third leading cause of death after cardiovascular and neoplastic diseases, with a prevalence of 6.5% [4]. Between 2004 and 2010, the incidence of stroke in rural China was significantly higher than in urban China and Western countries [5]. The “Brief report on stroke prevention and treatment in China, 2020” mentioned that stroke is the leading cause of death and disability among adults in China. The standardized prevalence of stroke among people over 40 years old in China increased from 1.89% in 2012 to 2.58% in 2019, and the burden of stroke disease is also increasing [6].

In China, vegetable intake declined significantly from 1991 to 2011 [7]. The reasons for this phenomenon may be related to the social situation, economic reforms in China, and a more diverse diet [8,9,10]. Data from 2010 to 2012 showed the average daily consumption of vegetables for Chinese residents aged over 33 was (255 ± 6) g/d [11]. In 2015, the intake of vegetables for adults aged 60 and above was 270 g/d [12]. According to the Dietary Guidelines for Chinese Residents, the consumption among Chinese adults was lower than the recommended daily consumption of 300–500 g of vegetables [13].

Vegetables have been a cornerstone of healthy dietary recommendations around the world. Vegetables are rich in dietary fiber and vitamins and provide a variety of phytochemicals that are beneficial to the body’s health, such as vitamin C, vitamin E, β-carotene, and other antioxidants such as selenium, zinc, and flavonoids. Dark vegetables are especially rich in carotene [14]. Vegetables are an important potential advantageous factor of most chronic diseases in Western societies [15,16,17,18,19,20,21]. In previous studies, there have also been articles discussing the relationship between vegetable consumption and stroke in other countries. A recent meta-analysis showed vegetable intake was associated with a reduced risk of cardiovascular disease, cancer, and all-cause mortality [16]. However, in a Japanese study, the consumption of vegetables, as well as Okinawan vegetables, was associated with stroke and coronary heart disease. The multivariable-adjusted hazard ratios of the highest versus the lowest in consumption were 1.09 (95% confidence interval, 0.93–1.29) [22]. Coincidentally the same findings were found in a Danish study, they found the intake of vegetables cannot reduce the risk of ischemic stroke [23]. On the other hand, some studies have shown that the consumption of vegetables can reduce the risk of stroke [24,25,26]. Particularly, the intake of green leafy vegetables was inversely associated with stroke [25,27,28]. Whether there is an association between vegetable consumption and stroke is debated, and the specific link between the two is unclear. In the current paper, we aim to discuss the relationship between vegetable intake and stroke risk in Chinese residents.

## 2. Materials and Methods

### 2.1. Study Population

Participants for this study were from the China Health and Nutrition Survey (CHNS), which is a cohort study with long-term follow up. The survey began in 1989 and used stratified multistage cluster sampling in China. The initial objective of the investigation was to determine the impact of Chinese society’s development on the residents’ health and nutritional status.

**Figure 1:**
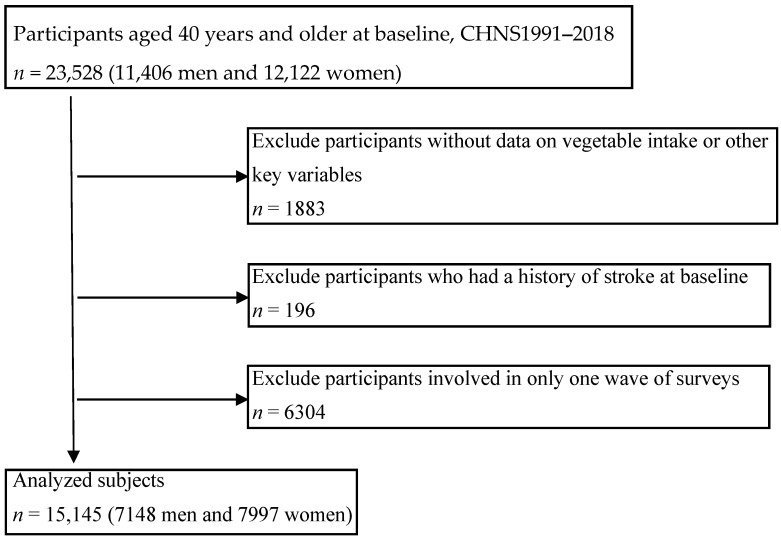
Flowchart for the selection of analyzed cohort.

This study used data from ten waves of CHNS conducted in 1991, 1993, 1997, 2000, 2004, 2006, 2009, 2011, 2015, and 2018. A total of 23,528 participants (11,406 men and 12,122 women), aged 40 years old and above were included at the baseline (Figure 1). First, we excluded 1883 persons without data on vegetable intake or other key variables. Secondly, we ruled out 196 participants with a stroke history at baseline. Then we excluded 6304 persons who just participated in only one wave of surveys. Finally, 15,145 participants (7148 men and 7997 women) were analyzed in the current study.

### 2.2. Vegetable Intake and Other Measurements

The dietary vegetable data in this survey were obtained from consecutive 24 h dietary recalls (2 weekdays and 1 weekend day) [29,30]. We classified vegetables into dark vegetables (carotene content > 500 μg/100 g), light vegetables (carotene content ≤ 500 μg/100 g), and pickles [31]. The classification of vegetables is based only on their carotene content, regardless of color. According to the quintiles of vegetable consumption, we classified participants into five groups Q1 (0~212 g/d), Q2 (213~271 g/d), Q3 (272~325 g/d), Q4 (326~398 g/d), and Q5 (≥399 g/d). We classified dark vegetable consumption groups into Q1 (0~25 g/d), Q2 (26~51 g/d), Q3 (52~82 g/d), Q4 (83~125 g/d), and Q5 (≥126 g/d), according to the quintile values.

In addition to dietary data, the baseline data of subjects included interviewed questionnaires and physical measurements. In this study, we collected the basic information about the participants through questionnaires, including age, gender, education level (primary, middle, high, and above), geographic region, smoking, alcohol consumption, annual average per capita household income (<1364.3, 1364.3–2728.6, and >2728.6 EUR) [32], physical activity (<95, 95–239, >239 met·hours/week; grouping by the tertiles of data distribution), the average MET hours per week measurements incorporated both the time spent on each activity and the average intensity of each activity [33,34]. History of illness included diabetes and hypertension (these diseases occurred before the study participants were investigated). Information on disease history was obtained through questionnaires (“Has a doctor ever told you that you suffer from diabetes?” “Has a doctor ever told you that you suffer from hypertension?”) We acquired the height, weight, SBP (systolic blood pressure), and DBP (diastolic blood pressure) information about the person by physical measurements. BMI groups are divided into four groups based on criteria applicable to the Chinese population, <18.5 kg/m^2^, 18.5~23.9 kg/m^2^, 24.0~27.9 kg/m^2^, and ≥28.0 kg/m^2^ [35]. Dietary data are the means of several rounds of investigation, and other data are from the baseline. The dietary data include vegetable consumption, dark vegetable consumption, energy, fat, cholesterol, meat, fish, fruit, and sodium intake.

The outcome event for this study was the participant had a stroke, obtained by the question “Has a doctor ever given you the diagnosis of stroke or transient ischemic attack?”.

### 2.3. Statistical Analysis

In this study, we stratified all analyses by gender and achieved statistical analysis with SAS 9.4 software. In the analysis of the baseline data, we used a one-way analysis of variance (ANOVA) for continuous variables and a chi-square test for categorical variables. We used the Kolmogorov–Smirnov test to verify the normality of continuous data. If the variables do not conform to a normal distribution, then the Wilcoxon rank-sum test was used for analysis. The relationship between vegetable intake and stroke was explored by a Cox proportional hazards model. The lowest quintile group was considered a reference to create 4 models: (1) was a crude model; (2) adjusted to baseline characters, such as age, gender, education level, working status, geographic region, and average household income; (3) adjusted to baseline characters and dietary factors (average values), such as fat, cholesterol, meat, fish, fruit, sodium, and total energy intake; and (4) further adjusted to baseline characters, dietary factors (average values), physical activity, BMI, smoking status, alcohol consumption, SBP, DBP, and a history of diabetes and hypertension.

## 3. Results

A series of 15,145 participants were included in this research, including 7148 males and 7997 females. The basic pieces of information were presented (Table 1). Vegetable consumption was classified into five levels: 0~212 g/d, 213~271 g/d, 272~325 g/d, 326~398 g/d, and ≥399 g/d. The amounts (percentages) of men in the vegetable consumption categories were 1234 (17.3%), 1327 (18.6%), 1407 (19.7%), 1504 (21.0%), and 1676 (23.4%), respectively. The amounts (percentages) of women were 1795 (22.4%), 1702 (21.3%), 1622 (20.3%), 1525 (19.1%), and 1353 (16.9%), respectively. The median age of all males was 48.9 years. Most of the males completed primary school (41.2%), lived in rural areas (60.2%), and were working employed (71.2%). Males who consumed vegetables more had lower BMI, higher energy, fat intake, higher meat, fish, dark vegetable, sodium intake, and lower fruit intake. The situation for women was broadly consistent with that of men, but among women, high vegetable intake is accompanied by high fruit intake. The number of subjects with diabetes and hypertension was lower in the high vegetable intake group compared to the low vegetable intake group.

From 1991 to 2018, there were fluctuating changes in the consumption of various types of vegetables from an overall perspective. The vegetable consumption decreased from 346.36 g/d to 268.86 g/d. The vegetable consumption among males decreased from 357.31 g/d to 278.98 g/d, while that among females decreased from 336.48 g/d to 260.49 g/d. From 1991 to 2018, dark vegetable consumption decreased from 93.23 g/d to 58.14 g/d. Dark vegetable consumption among males decreased from 95.12 g/d in 1991 to 58.81 g/d, while that decreased from 91.52 g/d to 57.58 g/d among females. (Supplementary Materials).

The total number of person-years of follow up in this survey was 150,590 and the mean observational period was 9.9 years. During the follow up period, stroke occurred in 504 (303 men and 201 women) subjects. Table 2 shows the relationship between different levels of vegetable consumption and stroke, which were obtained by adjusted models with HRs (95%CIs). Compared with the Q1 group, the multivariable-adjusted HRs (Model 4) for vegetable consumption were 1.16 (95%CI 0.86, 1.57) in the Q2 group, 1.17 (95%CI 0.86, 1.58) in the Q3 group, 0.94 (95%CI 0.68, 1.29) in the Q4 group, and 0.89 (95%CI 0.63, 1.24) in the Q5 group. For the males, compared with the Q1 group, the multivariable-adjusted HRs (Model 4) for vegetable consumption were 1.38 (95%CI 0.90,2.11) in the Q2 group, 1.31 (95%CI 0.85, 2.01) in the Q3 group, 1.24 (95%CI 0.80, 1.92) in the Q4 group, and 1.11 (95%CI 0.70, 1.75) in the Q5 group. For the females, compared with the Q1 group, the multivariable-adjusted HRs (Model 4) for vegetable consumption were 0.99 (95%CI 0.65, 1.53) in the Q2 group, 0.99 (95%CI 0.64, 1.53) in the Q3 group, 0.60 (95%CI 0.36, 1.00) in the Q4 group, and 0.60 (95%CI 0.35, 1.04) in the Q5 group (Table 2).

Table 3 shows the relationship between different levels of dark vegetable consumption and stroke, which were obtained by adjusted models with HRs (95%CIs). Compared with the Q1 group, subjects consuming 83~125 g/d had significantly lower risks in all models. The multivariable-adjusted HRs (Model 4) for dark vegetable consumption were 0.68 (95%CI 0.50, 0.92). For the males, no significant associations were found between dark vegetable consumption and stroke. For the females, compared with the Q1 group, subjects consuming 83~125 g/d had significantly lower risks in all models. The multivariable-adjusted HRs (Model 4) for dark vegetable consumption were 0.49 (95%CI 0.30, 0.80) (Table 3).

## 4. Discussion

In the CHNS study cohort, we found that women in China who consumed vegetables at the consumption of 326~398 g/d may tend to have a decreased risk of stroke compared with those who consumed 0~212 g/d. But we found no significant association between the consumption of vegetables and the incidence of stroke among men. In addition, adults with 83~125 g/d of dark vegetable consumption had a lower risk of stroke.

The findings of the previous studies about the association between vegetable consumption and stroke have been inconsistent [23,24,27,28,36,37]. In a prospective cohort study, the result displayed a protective relationship between the consumption of vegetables and ischemic stroke risk, particularly cruciferous and green leafy vegetables [27]. The Framingham Study of men aged 45 to 65 years, found vegetables may protect against the development of stroke in men [36]. A Danish prospective cohort study found an increased intake of vegetables may reduce the risk of ischemic stroke [23]. A study in the Netherlands found that a high intake of raw vegetables may protect against stroke. But no association was found between processed vegetable consumption and incident stroke [24]. A Swedish study analyzed an inverse association of vegetable consumption with stroke risk. Particularly, the consumption of green leafy vegetables was inversely associated with stroke [16]. In America, a study analyzed that vegetable consumption (≥3 times/d) was associated with a 27% lower stroke incidence (RR = 0.73, CI: 0.57–0.95), and showed an inverse association of vegetable intake with the risk of stroke [37]. However, other studies regarding the association between vegetable consumption and the risk of stroke found a nonsignificant association. A cohort study aimed to explore the association of vegetable and Okinawan vegetable consumption with the risk of incident stroke among Japanese residents aged 45–74 years, and found that total vegetables and Okinawan vegetables were not associated with the risk of incident stroke [22]. In a Netherlands study, the results showed that, independently of quantity, variety in vegetables was not related to incident stroke [38]. In addition, the Atherosclerosis Risk in Communities (ARIC) cohort was used to illustrate the association between vegetable intake and ischemic stroke among participants aged 45–64 years, which showed vegetable consumption did not have an association with the risk of ischemic stroke [39]. The different results of previous studies may be related to the diversity of diets in different national regions, and the fact that vegetable consumption and stroke incidence are not entirely consistent, all of which may lead to different results. Our results indicated that the proportion of those who consumed 326~398 g/d of vegetables, and 83~125 g/d of dark vegetables among males, was lower than that among females. Moreover, a higher proportion of men had stroke risk factors, such as hypertension, and diabetes, compared to women. These factors might have a stronger impact on the incidence of stroke than vegetable consumption, which might have masked the association between vegetable intake and the incidence of stroke. In addition, the determination of stroke outcomes in the study was based on participants’ self-reported questionnaires. It leads to incomplete information, which causes a lower number of stroke cases and undermines research into the association between vegetable consumption and stroke. Stroke events, diabetic events, and hypertensive events in this database are based on self reporting and no cause-of-death investigations are reported, which may be influenced by recall bias. There may be some inaccuracies in the data due to the lack of information on stroke deaths.

The exact mechanism by which moderated vegetable consumption leads to a lower risk of stroke remains unclear, and there are several possible explanations. Nutrients included in vegetables might have played a critical role in reducing the incidence of stroke. First, flavonoids are ubiquitously present in fruits and vegetables [40], which was associated with a reduction in the risk of ischemic stroke [41,42,43]. In addition, vegetables supply dietary fiber [44]. It is noteworthy that the intake of dietary fiber, especially vegetable fibers, is inversely associated with the risk of stroke [45,46]. Dietary fiber has several potential mechanisms that can reduce the risk of stroke. Hypertension is an important risk factor for stroke. The protective effect of dietary fiber on stroke may be partially related to its blood pressure-lowering effect. It can be seen that the health effect of dietary fiber on the body cannot be ignored [47]. Third, vegetables are also a major source of folate and vitamin B_6_ [48,49], which were found associated with reduced risk of mortality from stroke among the Japanese [43]. On the other hand, from the perspective of vegetable types, although some studies believe that it is not related to stroke [38], some studies have concluded that eating cruciferous and green leafy vegetables can reduce the risk of stroke [27]. The possible involvement of oxidative damage and antioxidant protection has been suggested in the pathogenesis of stroke [50]. Dark vegetables are rich in β-carotene, and a systematic review and a meta-analysis have shown that β-carotene significantly decreased the risk of stroke [51]. The study found that plasma concentrations of alpha- and beta-carotene were lower in acute ischemic stroke patients than in healthy controls and were negatively associated with neurological deficits, which the authors attributed to the antioxidant mechanism of carotenoids [52]. Furthermore, β carotene that is converted to vitamin A in the human body is a free radical scavenger and deletes singlet oxygen, superoxide, and hydroxyl radicals [53], and in this way prevents the risk of stroke. Most studies have reported reduced carotenoid levels after stroke [50,52]. Increased carotenoid depletion by oxidative stress may be one of the reasons for the decrease in carotenoids.

### Strengthens and Limitations

The advantages of this study include data based on Chinese cohorts and long-term follow-up studies and gender-specific discussions of the effects of vegetable consumption on stroke. In addition, the subjects who entered the cohort were strictly controlled, and the basic information on the individual, such as age, gender, education level, urban and rural distribution, etc., were controlled in the survival analysis model, and the factors such as meat, fruit, fat intake, and total energy in the diet were adjusted, and the influence of confounding factors was strictly controlled. The accuracy of the study was greatly improved. Of course, the study also has the following limitations. First, this population cohort mainly studies dietary changes in China. Compared to the traditional cardiovascular cohort, our results may underestimate the number of strokes, leading to skewed results. Second, the questionnaires collected during follow-up showed whether a stroke occurred only from the self-report of the study participants, and lack of detailed medical diagnosis. In addition, since the information is incomplete, it cannot greatly distinguish between the type of stroke (ischemic stroke or hemorrhagic stroke). From this, it appears that different types of vegetables may also be potentially linked to different stroke types.

## 5. Conclusions

In this long-term follow-up cohort study in China, higher vegetable consumption may decrease the risk of stroke among females. In particular, the intake of more carotenoid-rich dark vegetables was inversely associated with stroke. Consuming more dark vegetables may be effective in preventing stroke in the Chinese population. The results provide more evidence for guidelines or health policies for the primary prevention of stroke. Further research needs to be carried out on vegetable consumption and different stroke types, and the potential mechanisms.

## Figures and Tables

**Table 1:** Baseline characteristics of study subjects by vegetable consumption groups (CHNS study, 1991–2015).

	Total	Vegetable Consumption (g/d)					*p*
		Q1	Q2	Q3	Q4	Q5	
Males (*n* = 7148)							
Number of subjects	7148	1234	1327	1407	1504	1676	
Age (years)	48.9(42.7, 59.2)	53.7(44.4, 63.0)	50.1(43.3, 60.8)	48.7(42.5, 59.1)	47.6(42.3, 57.5)	46.6(42.2, 55.7)	<0.001
Education level (%)							
Primary school	2944(41.2%)	424(34.4%)	482(36.3%)	588(41.8%)	628(41.8%)	822(49.1%)	<0.001
Middle school	2173(30.4%)	383(31.0%)	420(31.7%)	411(29.2%)	469(31.2%)	490(29.2%)	
High school and above	2031(28.4%)	427(34.6%)	425(32.0%)	408(29.0%)	407(27.1%)	364(21.7%)	
Geographic region (%)							<0.001
Urban	2842(39.8%)	652(52.8%)	618(46.6%)	585(41.6%)	546(36.3%)	441(26.3%)	
Rural	4306(60.2%)	582(47.2%)	709(53.4%)	822(58.4%)	958(63.7%)	1235(73.7%)	
Working status							<0.001
Employed	5105(71.2%)	728(59.0%)	900(67.8%)	1014(72.1%)	1118(74.3%)	1345(80.3%)	
Smoking	4680(65.5%)	803(65.1%)	857(64.6%)	929(66.0%)	977(65.0%)	1114(66.5%)	0.799
Drinking	4532(63.4%)	715(57.9%)	817(61.6%)	920(65.4%)	984(65.4%)	1096(65.4%)	<0.001
Average per capita household income (EUR/year)							<0.001
<1364.3	5223(73.1%)	716(58.0%)	896(67.5%)	1085(77.1%)	1174(78.1%)	1352(80.7%)	
1364.3–2728.6	977(13.7%)	241(19.5%)	214(16.1%)	163(11.6%)	170(11.3%)	189(11.3%)	
>2728.6	948(13.3%)	277(22.5%)	217(16.4%)	159(11.3%)	160(10.6%)	135(8.1%)	
Physical activity (met·hours/week)							<0.001
<95	2435(34.1%)	481(34.6%)	528(37.5%)	508(36.5%)	474(33.1%)	444(29.1%)	
95–239	2364(33.1%)	401(28.8%)	461(32.8%)	506(36.4%)	503(35.1%)	493(32.3%)	
>239	2349(32.9%)	510(36.6%)	418(29.7%)	377(27.1%)	456(31.8%)	588(38.6%)	
Total vegetable (g/day)	311.6(238.9, 391.7)	175.1(144.3, 194.8)	243.4(228.2, 258.3)	300.0(286.5, 313.3)	358.9(342.2, 376.8)	466.2(426.7, 524.1)	<0.001
Dark vegetable (g/day)	66.7(33.3, 116.7)	33.3(16.7, 59.0)	50.3(26.7, 80.6)	68.0(36.4, 106.4)	84.5(45.7, 131.6)	118.9(71.6, 175.2)	<0.001
Meat intake (g/day)	62.1(30.6, 100.0)	46.2(20.3, 77.9)	58.2(27.9, 91.7)	63.9(33.3, 97.0)	70.2(37.5, 108.6)	73.9(34.6, 113.1)	<0.001
Fish intake (g/day)	20.0(0.0, 50.2)	11.1(0.0, 40.0)	19.2(0.0, 50.0)	24.5(0.0, 53.9)	21.3(0.0, 50.1)	23.2(0.0, 57.7)	<0.001
Fruit intake (g/day)	13.3(0.0, 56.1)	11.1(0.0, 53.3)	17.9(0.0, 62.2)	16.7(0.0, 64.2)	16.2(0.0, 55.6)	6.2(0.0, 46.8)	0.002
Sodium intake (mg/day)	5203.6(4080.5, 6687.5)	4702.6(3565.6, 6154.7)	5044.0(3940.2, 6497.4)	5189.8(4128.2, 6584.2)	5236.1(4174.7, 6581.1)	5674.0(4538.1, 7223.9)	<0.001
Energy intake (kcal/day)	2359.6(1882.2, 2856.0)	2030.7(1626.1, 2499.9)	2240.3(1777.5, 2708.1)	2364.1(1905.6, 2831.1)	2433.3(1980.5, 2944.6)	2603.9(2141.7, 3120.0)	<0.001
Fat intake (g/day)	79.3(62.1, 99.6)	72.2(55.6, 90.3)	76.3(59.5, 94.8)	78.1(62.8, 97.9)	82.5(64.8, 102.2)	86.7(66.7, 107.4)	<0.001
Cholesterol intake (g/day)	244.4(152.6, 346.9)	221.9(128.5, 322.0)	242.9(156.1, 344.7)	241.8(163.9, 343.6)	260.8(161.8, 363.3)	249.9(153.2, 357.4)	<0.001
BMI (kg/m^2^)							<0.001
<18.5	353(4.9%)	65(5.3%)	53(4.0%)	67(4.8%)	76(5.1%)	92(5.5%)	
18.5~23.9	4091(57.2%)	616(49.9%)	697(52.5%)	824(58.6%)	896(59.6%)	1058(63.1%)	
24.0~27.9	2086(29.2%)	422(34.2%)	432(32.6%)	416(29.6%)	401(26.7%)	415(24.8%)	
≥28.0	618(8.7%)	131(10.6%)	145(10.9%)	100(7.1%)	131(8.7%)	111(6.6%)	
History of disease (%)							
Diabetes	201(2.8%)	55(4.5%)	45(3.4%)	31(2.2%)	38(2.5%)	32(1.9%)	<0.001
Hypertension	797(11.2%)	203(16.5%)	170(12.8%)	146(10.4%)	150(10.0%)	128(7.6%)	<0.001
Females (n = 7997)							
Number of subjects	7997	1795	1702	1622	1525	1353	
Age (years)	48.7(42.5, 59.1)	52.7(43.6, 63.0)	50.3(42.9, 60.7)	48.1(42.4, 58.1)	46.5(42.1, 56.5)	45.9(41.9, 54.3)	<0.001
Education level (%)							<0.001
Primary school	4565(57.1%)	891(49.6%)	949(55.8%)	930(57.3%)	928(60.9%)	867(64.1%)	
Middle school	1862(23.3%)	462(25.7%)	373(21.9%)	388(23.9%)	353(23.2%)	286(21.1%)	
High school and above	1570(19.6%)	442(24.6%)	380(22.3%)	304(18.7%)	244(16.0%)	200(14.8%)	
Geographic region (%)							<0.001
Urban	3261(40.8%)	938(52.3%)	762(44.8%)	678(41.8%)	515(33.8%)	368(27.2%)	
Rural	4736(59.2%)	857(47.7%)	940(55.2%)	944(58.2%)	1010(66.2%)	985(72.8%)	
Working status							<0.001
Employed	4345(54.3%)	759(42.3%)	852(50.1%)	909(56.0%)	931(61.1%)	894(66.1%)	
Smoking	390(4.9%)	86(4.8%)	110(6.5%)	94(5.8%)	64(4.2%)	36(2.7%)	<0.001
Drinking	915(11.4%)	176(9.8%)	174(10.2%)	201(12.4%)	185(12.1%)	179(13.2%)	0.008
Average per capita household income (EUR/year)							<0.001
<1364.3	5776(72.2%)	1066(59.4%)	1202(70.6%)	1236(76.2%)	1177(77.2%)	1095(80.9%)	
1364.3–2728.6	1142(14.3%)	339(18.9%)	264(15.5%)	185(11.4%)	201(13.2%)	153(11.3%)	
>2728.6	1079(13.5%)	390(21.7%)	236(13.9%)	201(12.4%)	147(9.6%)	105(7.8%)	
Physical activity (met·hours/week)							<0.001
<95	2611(32.7%)	586(35.8%)	558(34.4%)	552(33.7%)	526(32.9%)	389(25.9%)	
95–239	2685(33.6%)	518(31.6%)	572(35.3%)	606(37.0%)	516(32.3%)	473(31.5%)	
>239	2701(33.8%)	533(32.6%)	492(30.3%)	480(29.3%)	555(34.8%)	641(42.7%)	
Total vegetable (g/day)	287.1(220.5, 363.1)	170.8(140.2, 193.0)	245.3(229.9, 258.8)	297.5(283.9, 311.0)	358.0(341.7, 376.6)	463.9(424.3, 518.7)	<0.001
Dark vegetable (g/day)	65.5(32.5, 110.3)	33.3(16.2, 56.7)	54.4(29.0, 86.0)	68.9(40.7, 105.8)	89.5(51.0, 131.7)	122.5(76.0, 183.4)	<0.001
Meat intake (g/day)	50.1(24.1, 82.3)	34.8(15.0, 61.9)	47.1(23.3, 77.2)	56.0(28.2, 84.0)	58.7(31.3, 92.3)	60.5(29.2, 98.6)	<0.001
Fish intake (g/day)	16.7(0.0, 41.7)	9.0(0.0, 33.3)	16.7(0.0, 41.7)	16.7(0.0, 40.2)	19.0(0.0, 46.3)	19.6(0.0, 50.0)	<0.001
Fruit intake (g/day)	23.0(0.0, 71.3)	25.0(0.0, 76.9)	28.0(0.0, 77.8)	25.3(0.0, 75.0)	20.0(0.0, 64.7)	13.3(0.0, 57.5)	<0.001
Sodium intake (mg/day)	4573.7(3543.1, 5918.0)	4058.5(3071.5, 5357.7)	4447.2(3487.5, 5775.2)	4543.3(3563.4, 5797.5)	4722.1(3768.3, 6064.6)	5235.6(4100.0, 6627.4)	<0.001
Energy intake (kcal/day)	1991.2(1574.6, 2462.8)	1690.2(1363.4, 2135.1)	1919.5(1534.8, 2383.1)	2021.2(1649.9, 2441.3)	2122.8(1673.7, 2589.0)	2264.1(1857.9, 2746.9)	<0.001
Fat intake (g/day)	68.2(53.4, 85.6)	61.0(47.7, 76.6)	65.0(51.5, 82.2)	69.3(54.5, 85.2)	71.8(58.4, 89.0)	76.5(58.3, 94.9)	<0.001
Cholesterol intake (g/day)	211.0(128.9, 307.9)	194.4(110.1, 292.6)	211.1(134.3, 304.4)	214.7(134.7, 306.3)	221.5(137.5, 317.5)	216.0(127.5, 325.2)	<0.001
BMI (kg/m^2^)							<0.001
<18.5	424(5.3%)	96(5.4%)	84(4.9%)	92(5.7%)	72(4.7%)	80(5.9%)	
18.5~23.9	4246(53.1%)	867(48.3%)	873(51.3%)	874(53.9%)	846(55.5%)	786(58.1%)	
24.0~27.9	2486(31.1%)	610(34.0%)	565(33.2%)	485(29.9%)	453(29.7%)	373(27.6%)	
≥28.0	841(10.5%)	222(12.4%)	180(10.6%)	171(10.5%)	154(10.1%)	114(8.4%)	
History of disease							
Diabetes	213(2.7%)	66(3.7%)	48(2.8%)	28(1.7%)	37(2.4%)	34(2.5%)	0.01
Hypertension	941(11.8%)	275(15.3%)	211(12.4%)	196(12.1%)	141(9.3%)	118(8.7%)	<0.001

Abbreviations: Age, physical activity, total vegetable, dark vegetable, meat intake, fish intake, fruit intake, sodium intake, energy intake, fat intake, cholesterol intake. The value of this variable in the table is the median (interquartile range: P25, P75).

**Table 2:** Multivariate adjusted hazard ratios (HR) for stroke, according to vegetable consumption (CHNS study, 1991–2018).

	Vegetable Consumption(g/d)				
	Q1	Q2	Q3	Q4	Q5
Total					
Number of participants	3029	3029	3029	3029	3029
Person years	19,793	29,200	32,753	33,777	35,067
Number of cases	70	120	125	99	90
HR (95%CI)					
Model 1	1	0.93(0.69, 1.25)	0.82(0.61, 1.10)	**0.62(0.45, 0.84)**	**0.52(0.38, 0.71)**
Model 2	1	1.14(0.84, 1.53)	1.10(0.82, 1.49)	0.90(0.65, 1.23)	0.78(0.56, 1.09)
Model 3	1	1.18(0.87, 1.59)	1.16(0.85, 1.57)	0.95(0.69, 1.31)	0.84(0.60, 1.18)
Model 4	1	1.16(0.86, 1.57)	1.16(0.85, 1.57)	0.93(0.67, 1.28)	0.87(0.62, 1.22)
Males					
Number of participants	1234	1327	1407	1504	1676
Person years	7624	12,108	14,800	16,480	19,224
Number of cases	32	67	71	69	64
HR (95%CI)					
Model 1	1	1.01(0.66, 1.54)	0.82(0.54, 1.26)	0.70(0.46, 1.07)	**0.53(0.35, 0.82)**
Model 2	1	1.32(0.86, 2.02)	1.16(0.76, 1.78)	1.13(0.73, 1.74)	0.91(0.58, 1.42)
Model 3	1	1.41(0.92, 2.16)	1.27(0.83, 1.95)	1.26(0.81, 1.94)	1.06(0.67, 1.66)
Model 4	1	1.38(0.90, 2.11)	1.31(0.85, 2.01)	1.24(0.80, 1.92)	1.11(0.70, 1.75)
Females					
Number of participants	1795	1702	1622	1525	1353
Person years	12,169	17,092	17,953	17,297	15,843
Number of cases	38	53	54	30	26
HR (95%CI)					
Model 1	1	0.81(0.53, 1.23)	0.75(0.49, 1.14)	**0.42(0.26, 0.69)**	**0.39(0.23, 0.64)**
Model 2	1	0.98 (0.64, 1.49)	1.06(0.69, 1.63)	0.64(0.39, 1.06)	0.66(0.39, 1.11)
Model 3	1	0.96(0.63, 1.48)	1.03(0.66, 1.59)	**0.61(0.37, 1.00)**	0.59(0.35, 1.03)
Model 4	1	0.99(0.65, 1.53)	0.99(0.64, 1.53)	**0.60(0.36, 1.00)**	0.60(0.35, 1.04)

Model 1 was a crude model. Model 2 was adjusted for age, gender, education level, working status, geographic region, and average household income, characteristics derived from the baseline. Model 3 was adjusted by variables in Model 2 plus fat, cholesterol, meat, fish, fruit, total energy, and sodium. Model 4 was adjusted by variables in model 3 plus physical activity BMI, smoking, drinking, history of diabetes, and hypertension. The bold is used to indicate statistically significant results.

**Table 3:** Multivariate adjusted hazard ratios (HR) for stroke, according to dark vegetable consumption (CHNS study, 1991–2018).

	Dark Vegetable Consumption (g/d)				
	Q1	Q2	Q3	Q4	Q5
Total					
Number of participants	3029	3029	3029	3030	3028
Person-years	23,839	29,707	30,727	33,171	33,145
Number of cases	90	110	103	90	111
HR (95%CI)					
Model 1	1	0.89(0.68, 1.18)	0.78(0.59, 1.03)	**0.61(0.45, 0.82)**	**0.75(0.56, 0.99)**
Model 2	1	0.85(0.64, 1.13)	**0.74(0.55, 0.98)**	**0.60(0.45, 0.81)**	0.79(0.59, 1.04)
Model 3	1	0.87(0.66, 1.16)	0.77(0.57, 1.02)	**0.64(0.47, 0.87)**	0.85(0.63, 1.14)
Model 4	1	0.89(0.67, 1.18)	0.80(0.60, 1.07)	**0.68(0.50, 0.92)**	1.01(0.75, 1.36)
Males					
Number of participants	1392	1407	1391	1433	1525
Person years	10,923	13,598	13,541	15,385	16,789
Number of cases	52	63	58	59	71
HR (95%CI)					
Model 1	1	0.89(0.62, 1.29)	0.80(0.55, 1.16)	**0.68(0.47, 0.99)**	0.73(0.51, 1.05)
Model 2	1	0.83(0.57, 1.20)	0.70(0.48, 1.02)	**0.66(0.46, 0.96)**	0.77(0.54, 1.10)
Model 3	1	0.88(0.60, 1.27)	0.76(0.52, 1.12)	0.75(0.51, 1.11)	0.91(0.62, 1.32)
Model 4	1	0.90(0.62, 1.30)	0.82(0.56, 1.20)	0.81(0.55, 1.19)	1.10(0.75, 1.60)
Females					
Number of participants	1637	1622	1638	1597	1503
Person years	12,916	16,110	17,186	17,786	16,356
Number of cases	38	47	45	31	40
HR (95%CI)					
Model 1	1	0.91(0,59, 1.39)	0.78(0.51, 1.21)	**0.51(0.31, 0.81)**	0.71(0.46, 1.11)
Model 2	1	0.89(0.58, 1.37)	0.78(0.50, 1.21)	**0.51(0.32, 0.82)**	0.80(0.51, 1.26)
Model 3	1	0.85(0.55, 1.32)	0.74(0.47, 1.15)	**0.47(0.28, 0.76)**	0.72(0.45, 1.16)
Model 4	1	0.88(0.57, 1.36)	0.72(0.46, 1.13)	**0.49(0.30, 0.80)**	0.82(0.50, 1.33)

Model 1 was a crude model. Model 2 was adjusted for age, gender, education level, working status, geographic region, and average household income, characteristics derived from the baseline. Model 3 was adjusted by variables in Model 2 plus fat, cholesterol, meat, fish, fruit, total energy, and sodium. Model 4 was adjusted by variables in model 3 plus physical activity, BMI, smoking, drinking, history of diabetes, and hypertension. The bold is used to indicate statistically significant results.

## Data Availability

The datasets generated and analyzed during the current study are available from the corresponding author (W.D.) upon reasonable request.

## Supplementary Materials

The following supporting information can be downloaded at: https://www.mdpi.com/article/10.3390/nu15071583/s1, Figure S1: Changes in vegetable consumption from 1991 to 2018.
