# Supplementary material for: Association of Vegetable Consumption with Stroke in China: A Longitudinal Study
Source: Nutrients. 2023 Mar 24;15(7):1583. doi: 10.3390/nu15071583 (PMC10096659; doi:10.3390/nu15071583)
Supplement: Supplementary file 1 [file nutrients-15-01583-s001.zip › nutrients-2287273-supplementary.docx]

**Figure S1.** Changes in vegetable consumption from 1991 to 2018.
